# Indoleamine 2,3-dioxygenase 1 (IDO1) inhibitors in clinical trials for cancer immunotherapy

**DOI:** 10.1186/s13045-021-01080-8

**Published:** 2021-04-21

**Authors:** Kai Tang, Ya-Hong Wu, Yihui Song, Bin Yu

**Affiliations:** 1 School of Pharmaceutical Sciences and Key Laboratory of Advanced Drug Preparation Technologies, Ministry of Education, Zhengzhou University, Zhengzhou, 450001 China; 2 School of Life Sciences, Zhengzhou University, Zhengzhou, 450001 China; 3 State Key Laboratory of Natural Medicines, China Pharmaceutical University, Nanjing, 210009 China

**Keywords:** Immune escape, IDO1 inhibitors, PROTAC degraders, Cancer therapy

## Abstract

Indoleamine 2,3-dioxygenase 1 (IDO1) is a heme enzyme that catalyzes the oxidation of *L*-tryptophan. Functionally, IDO1 has played a pivotal role in cancer immune escape via catalyzing the initial step of the kynurenine pathway, and overexpression of IDO1 is also associated with poor prognosis in various cancers. Currently, several small-molecule candidates and peptide vaccines are currently being assessed in clinical trials. Furthermore, the “proteolysis targeting chimera” (PROTAC) technology has also been successfully used in the development of IDO1 degraders, providing novel therapeutics for cancers. Herein, we review the biological functions of IDO1, structural biology and also extensively summarize medicinal chemistry strategies for the development of IDO1 inhibitors in clinical trials. The emerging PROTAC-based IDO1 degraders are also highlighted. This review may provide a comprehensive and updated overview on IDO1 inhibitors and their therapeutic potentials.

## Introduction

Normally, the immune system recognizes and obliterates foreign invaders including tumor cells in the tumor microenvironment [1]. However, tumor cells could avoid destruction by the immune system through multiple local immunosuppression mechanisms, which frequently contribute to the survival of tumor cells in the different stages of the anti-tumor immune response [2–4]. By reinstating the cancer-immunity cycle, cancer immunotherapy could strengthen the anti-tumor immune response, and restore the functions of the immune system to distinguish and eliminate tumor cells [5]. Currently, many immunotherapy therapies, including checkpoint inhibitors [6], monoclonal antibodies (mAbs) [7] and vaccines [8], are being developed for treating various cancers, especially melanoma, breast cancer and non-small cell lung cancer (NSCLC), etc. It has been widely recognized that combination of these novel therapies with standard chemo or radiotherapy could be an effective approach to overcome tumor-induced immunosuppression in clinic settings [9–11].

**Fig. 1:**
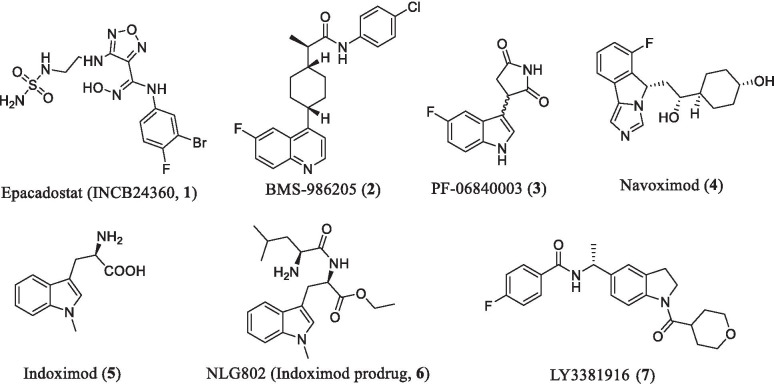
IDO1 inhibitors in the clinical stage

Previous studies have revealed that the kynurenine (Kyn) pathway is involved in tumor-associated immunosuppression [12]. In this pathway, three heme-containing enzymes including indoleamine 2,3-dioxygenase 1 (IDO1), indoleamine 2,3-dioxygenase 2 (IDO2) [13], and tryptophan 2,3-dioxygenase (TDO) [14] could catalyze the rate-limiting step of tryptophan (Trp) metabolism. Among them, IDO1 has played a pivotal role in this pathway [15–17]. IDO1 catalyzes the initial oxidation of *L*-tryptophan (*L*-Trp) and induces the accumulation of kynurenine metabolites [18–20], which lead to the suppression of T-cell and are responsible for tumor cells to escape the monitoring and clearance of the immune system [15]. Preclinical studies also support that IDO1 overexpression is associated with poor prognosis in the majority of cancers [21]. Over the past decade, intense efforts have been made to developing IDO1 inhibitors, and numerous small-molecule IDO1 inhibitors have been reported, some of them such as Epacadostat [22], BMS-986205 [23], Indoximod [24, 25] and PF-06840003 [26], etc., are currently being studied in clinical trials (Fig. 1). In addition, the “proteolysis targeting chimera” (PROTAC) technique has also been employed to design PROTAC-based IDO1 degraders, which show promise for cancer immunotherapy [27]. In this review, we aim to provide a timely and updated summary on the structural biology and biological functions of IDO1 as well as cancer immunotherapy of IDO1 inhibitors in clinical trials. Emphasis will be placed on the IDO1 inhibitors and peptide vaccines in clinical trials as well as the medicinal chemistry strategies for the development of IDO1 inhibitors. The recently reported PROTAC degraders, limitations and challenges of IDO1 inhibitors for cancer immunotherapy are also discussed.

## Biological functions of IDO1

### The rate-limiting enzyme in the Kyn pathway of Trp metabolism

**Fig. 2:**
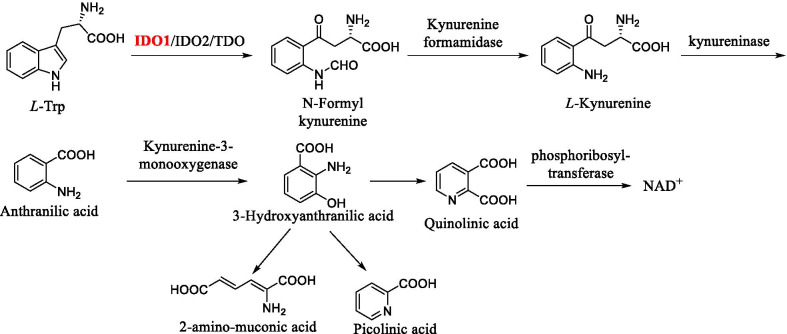
The Kyn pathway of Trp decomposition

The Kyn is an important metabolic pathway of Trp, which normally degrades Trp and generates corresponding catabolites. Different catabolites, involved in the formation of the immunosuppressive environment, are closely related to various diseases [28, 29]. As the first and rate-limiting step in the Kyn pathway of Trp decomposition (Fig. 2), IDO1 plays a key role in catalyzing the oxidative cleavage of Trp and regulating the level of Trp as well as corresponding metabolites in the body [30]. In the Kyn pathway, the pyrrole ring of *L*-Trp is catalytically oxidized by IDO1 to give *N*-formyl kynurenine, which is then converted to *L*-Kyn in the presence of kynurenine formamidase. Anthranilic acid is generated from *L*-Kyn in the presence of kynureninase and further converted to 3-hydroxyanthranilic acid by kynurenine-3-monooxygenase, finally generating three metabolites including 2-amino-muconic acid, picolinic acid, and quinolinic acid. Quinolinic acid subsequently activates the generation of nicotinamide adenine dinucleotide (NAD^+^) in the presence of phosphoribosyl-transferase.

### Expression, function of IDO1 and its relationship with tumor immune escape

IDO1 is an intracellular enzyme (principally in the cytosolic and perinuclear regions) to catalyze the conversion of *L*-Trp into Kyn. In normal physiological conditions, IDO1 is not expressed in many cell types and mainly expressed in mucosal tissues, placenta, eye, pancreas, and some immune cell subsets (some DC subsets, eosinophils) [28].

Studies have shown that relative to IDO2 and TDO, IDO1 has lower substrate specificity to catabolize Trp, and is associated with poor survival of various cancer patients [29, 31]. It has been reported that IDO1 is highly expressed in a variety of tumors, including colorectal cancer, breast cancer, esophageal carcinoma, cervical squamous cell carcinoma, melanoma, pancreatic cancer [32, 33], etc., and IDO1 is also expressed in many other cells, such as vascular cells, antigen-presenting cells (APCs, such as macrophages and DCs), eosinophils, endothelial cells (ECs), fibroblasts, and so on [34]. In addition, IDO1 can be inhibited by the cancer-suppression gene bridging integrator 1 (Bin1) and up-regulated by some cytokines and immune checkpoint molecules such as IFN-γ, prostaglandin E2 (PGE2), pathogen-associated molecular patterns (PAMPs, such as Toll-like receptor (TLR) 3, TLR4, TLR7, TLR8, and TLR9), damage-associated molecular patterns (DAMPs), immune checkpoint (including PD-1, glucocorticoid-induced TNF receptor-related protein (GITRL), CTLA-4), IL-6 and TNF-α, TGF-β to establish an immunosuppressive environment [33, 35].

There are two main Trp decomposition pathways concerned with IDO1-mediated tumor immune escape. A nuclear role has been attributed to the Kyn pathway of Trp metabolism, which leads to the consumption of Trp and generation of Kyn metabolites, both responsible for forming an immunosuppressive environment by suppressing the activation of effector cells and promoting the activation of immunosuppressive cells. IDO1 can influence the progression of tumors in three ways. Firstly, IDO1 promotes tumorigenesis and the formation of tolerogenic APCs to enhance the peripheral immune tolerance of tumor-associated antigens (TAAs) [36]. Secondly, overexpression of IDO1 in tolerogenic APCs can inhibit the proliferation and activity of CD8^+^ T effector cells (*T*_effs_) and NK cells but induce Tregs and MDSCs through the Kyn pathway indirectly [37]. IDO1 also promotes the expansion and activation of MDSCs and induces polarization of macrophages to a tolerogenic phenotype [38, 39]. Elevated CTLA4 expression of Tregs causes increased IDO1 secretion by DCs [40]. IDO1-induced expansion and activation of Tregs, MDSCs, and tolerogenic DCs suppress the activity of antitumor effector T cells [41, 42]. Finally, induced MDSCs could further inhibit the function of CD8^+^ T effector cells and NK cells through inflammation environment and induce cancer migration [43].

**Fig. 3:**
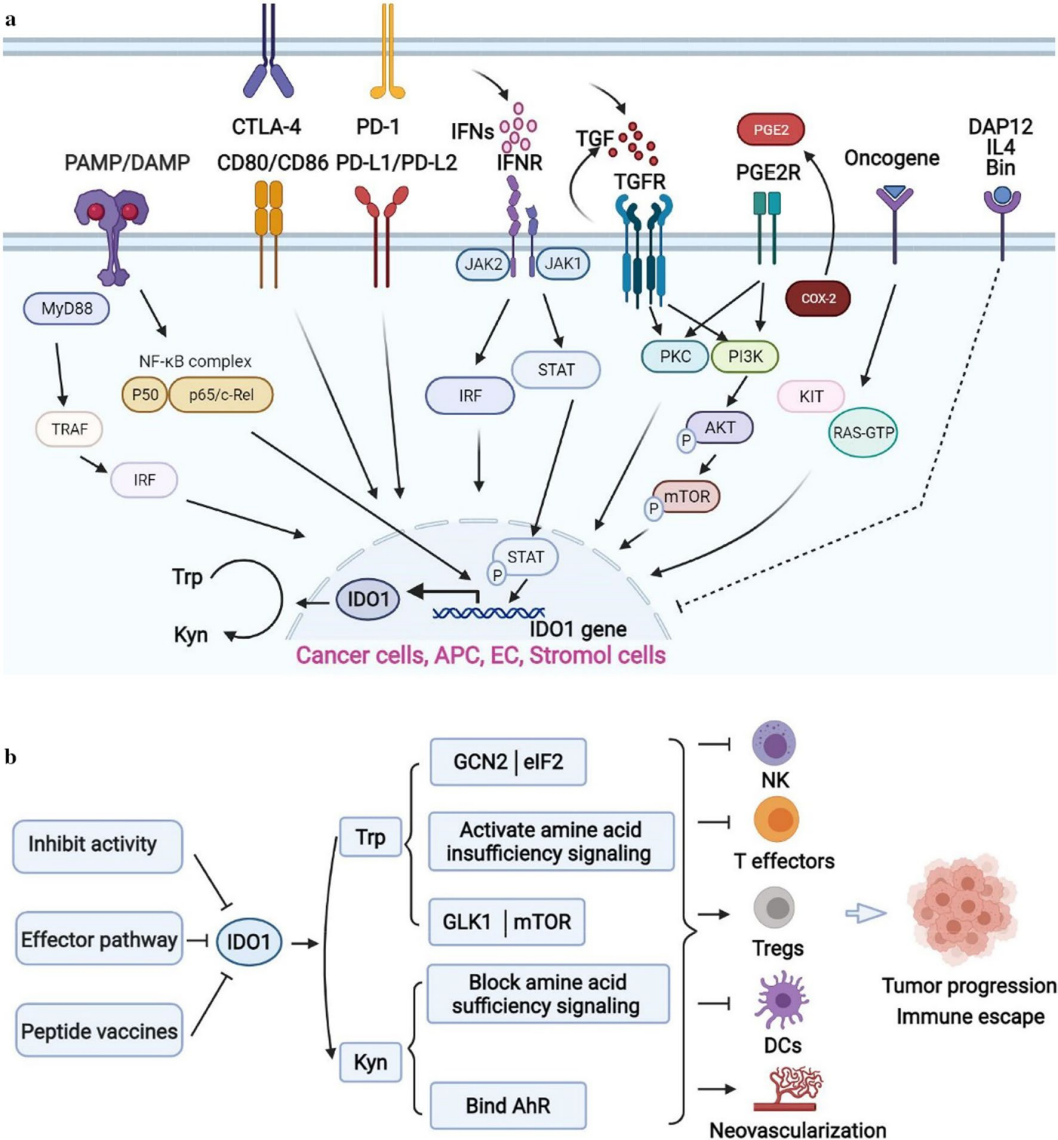
Mechanism of IDO1 regulation, function and activity. **a** Regulation of IDO1 expression in many cells through IFN-γ/JAK/STAT, KAS, NF-κB and PI3K pathways. **b** IDO1 targeting and mechanism of IDO1 in immune escape of cancer cells. IDO1 promotes the immune escape and progression of cancer mainly through GCN2/eIF2, GLK1/mTOR, AhR to inhibit the function and differentiation of effector T cells, and there are mainly three strategies to target IDO1 in clinical trials for cancer treatment

As shown in Fig. 3a, the expression and function of IDO1 can be regulated by inflammatory molecules (e.g. IFN-γ, TGF-β/PGE2, TNF-α, PAMPs/DAMPs) through multiple signaling pathways, such as Janus Kinase (JAK)/transducer and activator of transcription (STAT), NF-κB, phosphoinositide 3-kinase (PI3K), IFN regulatory factor 1 (IRF1), and RAS–PKC pathway [44, 45], etc. Cyclooxygenase 2 (COX-2) and PGE2 can drive the expression of IDO1 via the PKC and PI3K pathways [46]. In the promoter region of IDO1, there are two IFN-γ stimulated response elements (ISREs) and three IFN-γ activated sites (GASs), which interact with IRF1 and STAT1, respectively. Studies have shown that IFN-γ could potently induce the expression of IDO1, and could robustly activate the (JAK)/STAT and protein kinase Cδ (PKCδ) signaling. Accordingly, JAK, STAT1 and IRF1 play an important role in IFN-γ mediated IDO1 gene transcription. Autocrine TGF-β of a tolerogenic CD8^+^ DC could activate IDO1 [37]. Cancer suppression gene Bin1 could downregulate the expression of IDO1 via STAT1 and NF-κB dependent pathway [34]. IL-6 could upregulate the intestine-specific homeobox (ISX) gene to induce the expression of IDO1 and TDO, and promote the malignant potential of hepatocellular carcinoma cells [47]. In DCs, the expression of IDO1 can be up-regulated by the interaction of its surface CD80/86 with immune checkpoint molecules such as PD-1 and CTLA-4 expressed on the surface of Tregs. Bin1 could block the transcription of IDO1 which is mediated by STAT1 and NF-κB. Other cytokines such as tumor necrosis factor α (TNF-α), interleukin (IL)-1, IL-6, IFNα and IFNβ, or stimuli including muramyl tripeptide, glucocorticoids, and some infectious pathogens could also regulate the expression of IDO1 [44].

As shown in Fig. 3b, downstream signaling effectors of IDO1 are mainly general control over the mammalian target of rapamycin (mTOR), nonderepressible 2 (GCN2), and aryl hydrocarbon receptor (AhR) [44, 48]. Trp consumption by IDO1 could accumulate the uncharged Trp-tRNA to bind and activate GCN2, and then phosphorylates and inhibits eIF2α, leading to the attenuation of RNA transcription and protein translation. Activated GCN2 could induce the apoptosis or cell cycle arrest of effector T cells and also impacts the differentiation of CD4^+^ T cells. Another signaling molecule mTOR mediated by IDO1 can lead to anergy of T cells [30, 49]. It has been reported that Kyn is an endogenous ligand of AhR, increased Kyn levels activate the AhR that switches the activity of DCs from immunogenic to tolerogenic, and promotes CD4^+^ T cells differentiation into Treg cells [50, 51]. All these factors form an immunosuppressive tumor environment to promote the immune escape of tumor cells.

## Structural biology of IDO1

**Fig. 4:**
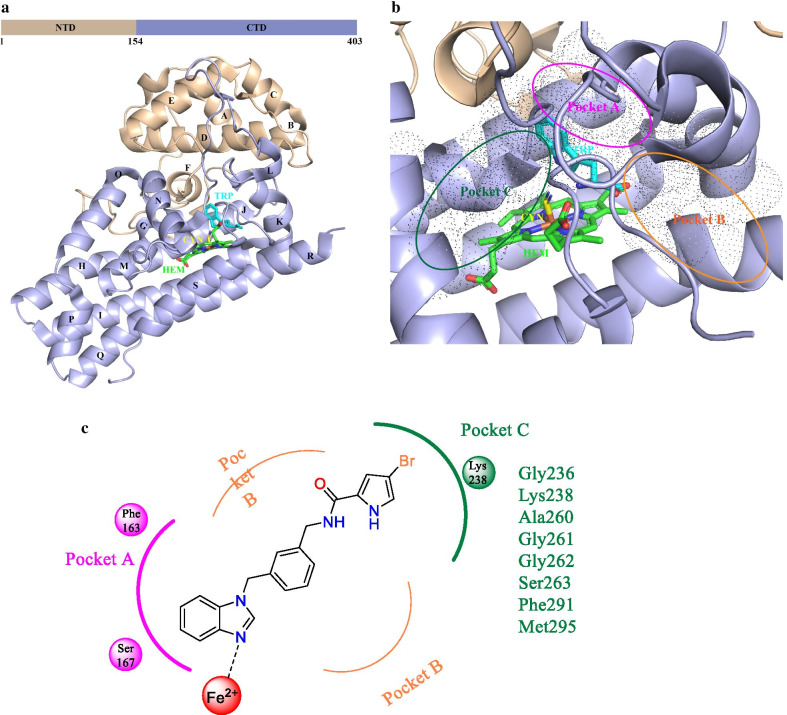
Structural basis of IDO1. **a** The overall structure of human IDO1 (PDB code: 5WMU). The N-terminal and C-terminal domains are represented by aurantium and purple ribbons, respectively. The helices A–S are named in the order of appearance in the primary sequence. The substrate Trp is colored in cyan and the heme site is colored in green. **b** Three binding pockets within the active site. The three pockets are colored in magentas (“Pocket A”), orange (“Pocket B”), and jasper (“Pocket C”), respectively. **c** Schematic representation of the binding model of the benzimidazole compound within the active site of IDO1

Human IDO1 (hIDO1) is an *α*-helical dioxygenase protein containing a small N-terminal domain (NTD, residues 1–154) and a large C-terminal domain (CTD, residues 155–403) (PDB code: 5WMU, Fig. 4a). The CTD is made up of thirteen α-helices (G-S) and two 3_10_ helices, four long helices (G, I, Q, and S) of which are ranged with the heme site (HEM) and form hydrophobic interactions with the neighboring helix, and the side chains of helices K, L, and N are also involved in heme–protein interactions [52–55]. Trp, the hIDO1’s binding substrate, induces the organization of the highly disordered JK-Loop of the CTD into a β-hairpin structure. These main helices create the heme-binding pocket together (“Pocket A”, Fig. 4b) [56, 57], the imidazole moiety of His346 in helix Q greatly contributes to the heme–protein interactions as a crucial endogenous ligand. While the NTD is located on top of it and consists of six α-helices, two short β-sheets, and three 3_10_ helices. There are extensive contacts between these two domains by a combination of numerous hydrophobic interactions and “salt bridges” [58]. Moreover, the “Pocket B” at the entrance of the active site is comprised of Phe226, Phe227, Arg231, Ile354, and Leu384, etc., and participates in stabilizing protein–inhibitor interactions [56, 59]. Mutants of Phe226, Phe227, and Arg231 have significantly reduced the dioxygenase activity, supporting the hypothesis that the residues of “Pocket B” directly affect substrate recognition by π–π stacking interactions and hydrophobic interactions. Additionally, the benzimidazole-based compound reported by Alberto Massarotti and co-workers [60, 61] has a unique binding mode within the active site of IDO1 (Fig. 4b, c). The 3-bromopyrrole moiety extends into an extra “Pocket C” consisting of Gly236, Lys238, Ala260, Gly261, Gly262, Ser263, Phe291, Met295, etc*.* Taken together, the structural features provide a basis for the structure-based design of IDO1 inhibitors.

## IDO1 inhibitors and peptide vaccines in clinical trials

Given the biological importance of IDO1 in cancer immunotherapy, IDO1 has become an attractive target. To date, a large number of IDO1 inhibitors have been reported, some of them, including Epacadostat, BMS986205, Indoximod, PF-06840003, etc., have advanced into clinical trials for cancer treatment (Fig. 1). Besides, several peptide vaccines targeting IDO1 have also entered the clinical assessment for examining the efficacy and safety and show promise for cancer therapy. Since the IDO1 inhibitors and peptide vaccines in clinical trials have been described previously in the decent review article [62], only recent updates on IDO1 inhibitors in clinical trials are shown in Table 1.

**Table 1:** IDO1 inhibitors in clinical trials

Drug	Strategy	Tumor type	Phase	Clinical efficacy	Safety	Trial number	Status
Indoximod	Single agent	Healthy	Phase I	Unknown	Unknown	NCT03372239	Completed
		Healthy	Early phase I	Unknown	Unknown	NCT03852446	Completed
		Progressive brain tumors or newly diagnosed DIPG	Phase II	Unknown	Unknown	NCT04049669	Recruiting
	Pembrolizumab/nivolumab	Advanced or metastatic melanoma	Phase I/II	Unknown	Unknown	NCT02073123	Completed
	Temozolomide	Refractory metastatic prostate cancer	Phase II	Stable disease (SD) 50%	Constipation, diarrhea, fatigue, pain,	NCT01560923	Completed
	Adenovirus-p53 transduced dendritic cell (DC) vaccine	Breast cancer	Not applicable	Unknown	Unknown	NCT01302821	Withdrawn
Epacadostat	Single agent	Epithelial ovarian, fallopian tube or primary peritoneal carcinoma	Early phase I	Unknown	Unknown	NCT02042430	Active, not recruiting
		Locally advanced rectal cancer	Phase I	Unknown	Unknown	NCT03516708	Recruiting
	Pembrolizumab	Gastrointestinal stromal tumors	Phase II	Unknown	Unknown	NCT03291054	Active, not recruiting
		Head and neck cancer	Phase II	Unknown	Unknown	NCT03238638	Withdrawn
		Squamous cell carcinoma of the head and neck	Phase II	Unknown	Unknown	NCT03325465	Withdrawn
		Head and neck cancer patients, who failed prior PD-1/PD-L1 therapy	Phase II	Unknown	Unknown	NCT03463161	Terminated
		Advanced pancreatic cancer	Phase II	Unknown	Unknown	NCT03432676	Withdrawn
		Recurrent Clear cell carcinoma of the ovary	Phase II	Unknown	Unknown	NCT03602586	Suspended
		Non-metastatic esophageal/gastroesophageal squamous cell and adenocarcinomas treated with neoadjuvant chemoradiation	Phase II	Unknown	Unknown	NCT03592407	Withdrawn
		Recurrent/metastatic endometrial carcinoma	Phase II	Unknown	Unknown	NCT03310567	Pembrolizumab
		Lung cancer	Phase II	Unknown	Unknown	NCT03322540	Active, not recruiting
		Urothelial cancer (UC)	Phase III	Unknown	Unknown	NCT03374488	Active, not recruiting
		Renal cell carcinoma (RCC)	Phase III	PR: 29.7%, CR + PR: 31.3%, [63]	Anaemia, diarrhoea, hypothyroidism, nausea, fatigue, pruritus	NCT03260894	Recruiting
		Muscle-invasive bladder cancer	Phase II	Unknown	Unknown	NCT03832673	Not yet recruiting
		Head and neck cancer	Phase III	Unknown	Hypothyroidism, vomiting, asthenia	NCT03358472	Active, not recruiting
		Extensive stage small cell lung carcinoma	Phase II	Unknown	Unknown	NCT03402880	Withdrawn
	Pembrolizumab/azacitidine	Metastatic colorectal cancer	Phase I/II	Unknown	Unknown	NCT03182894	Withdrawn
	Pembrolizumab/chemotherapy	Advanced solid tumors	Phase I	Unknown	Unknown	NCT02862457	Active, not recruiting
		Lung cancer	Phase II	Unknown	Nausea	NCT03322566	Active, not recruiting
	INCB001158/pembrolizumab	Solid tumors	Phase I/II	Unknown	Unknown	NCT03361228	Terminated
	Electroporation/pembrolizumab	Squamous cell carcinoma of the head and neck	Phase II	Unknown	Unknown	NCT03823131	Recruiting
	Nivolumab/Ipilimumab	Advanced or metastatic malignancies	Phase I/II	Unknown	Unknown	NCT03347123	Active, not recruiting
	Nivolumab/chemotherapy	Lung cancer	Phase III	Unknown	Unknown	NCT03348904	Terminated
		Nivolumab/chemotherapy	Phase III	Unknown	Unknown	NCT03342352	Withdrawn
	Durvalumab	Unresectable, recurrent, and metastatic EBV + NPC	Phase II	Unknown	Unknown	NCT04231864	Not yet recruiting
	Cladribine/cytarabine	Relapsed/refractory AML patients	Phase I	Unknown	Unknown	NCT03491579	Withdrawn
	Idarubicin/cytarabine/Daunorubicin	Phase 1	Phase I	Unknown	Unknown	NCT03444649	Withdrawn
	Rapamycin	Advanced malignancy	Phase I	Unknown	Unknown	NCT03217669	Recruiting
	INCMGA00012 + RT + bevacizumab	Recurrent gliomas	Phase II	Unknown	Unknown	NCT03532295	Recruiting
	M7824 + BN-Brachyury + ALT-803 + Epacadostat (Immunotherapy)	Solid tumor	Phase I/II	Unknown	Unknown	NCT03493945	Recruiting
	INCMGA00012, Epacadostat 600 mg BID, SV-BR-1-GM combination	Metastatic or locally recurrent breast cancer patients	Phase I/II	Unknown	Unknown	NCT03328026	Recruiting
	Intralesional SD101, Radiotherapy	Advanced solid tumors lymphoma	Phase I/II	Unknown	Unknown	NCT03322384	Recruiting
	Itacitinib/INCB050465	Solid tumors	Phase I	Unknown	Unknown	NCT02559492	Terminated
BMS-986205	Single agent	Healthy volunteers	Phase I	Unknown	Unknown	NCT03378310	Completed
						NCT03374228	
						NCT03312426	
						NCT03362411	
						NCT03247283	
	Nivolumab	Advanced malignant solid tumors	Phase I/ II	Unknown	Unknown	NCT03792750	Active, not recruiting
		Recurrent or persistent endometrial carcinoma or endometrial carcinosarcoma	Phase II	Unknown	Unknown	NCT04106414	Recruiting
		Squamous cell carcinoma of the head and neck	Phase II	Unknown	Unknown	NCT03854032	Recruiting
		Resectable stage III or IV melanoma	Phase II	Unknown	Unknown	NCT04007588	Withdrawn
	Relatlimab/nivolumab	Advanced malignant tumors	Phase I/ II	Unknown	Unknown	NCT03459222	Recruiting
	Nivolumab/BCG	BCG-unresponsive, high-risk, non-muscle invasive bladder cancer	Phase II	Unknown	Unknown	NCT03519256	Recruiting
	Itraconazole/rifampin	Malignancies multiple	Phase I	Unknown	Unknown	NCT03346837	Completed
	Nivolumab/chemotherapy	Muscle-invasive bladder cancer	Phase III	Unknown [64]		NCT03661320	Recruiting
	Nivolumab/radiotherapy or chemoradiotherapy	Glioblastoma	Phase I	Unknown	Unknown	NCT04047706	Recruiting
	Omeprazole	Healthy	Phase I	Unknown	Unknown	NCT03936374	Completed
Navoximod (GDC-0919, NLG-919)	Single agent	Recurrent advanced solid tumors	Phase I	8 (36%) had stable disease and 10 (46%) had progressive disease [65, 66]	Fatigue (59%), cough, decreased appetite, and pruritus (41% each), nausea (36%), and vomiting (27%). Grade ≥ 3 AEs occurred in 14/22 patients (64%)	NCT02048709	Completed
PF-06840003	Single agent	Malignant glioma	Phase I	Disease control occurred in eight patients (47%). Mean duration of stable disease (SD) was 32.1 (12.1–72.3) weeks [67, 68]	Grade 4 alanine and aspartate aminotransferase elevations	NCT02764151	Terminated
KHK2455	Avelumab	Urothelial carcinoma	Phase I	Unknown	Unknown	NCT03915405	Recruiting
	Mogamulizumab	Locally advanced or metastatic solid tumors	Phase I	Unknown	Unknown	NCT02867007	Active, not recruiting
LY3381916	Single agent or in combination with anti-programmed cell death ligand 1 (PD-L1) checkpoint antibody (LY3300054)	Solid tumor, non-small cell lung cancer, renal cell carcinoma, triple negative breast cancer	Phase I	Unknown	Unknown	NCT03343613	Terminated

### Indoximod (1-methyl-*D*-tryptophan, 1MT, NLG-8189)

Indoximod is a competitive IDO1 inhibitor and has been granted orphan-drug designation by the US FDA for the treatment of stage IIb to stage IV melanoma. Studies have reported that indoximod does not directly inhibit the enzymatic activity of IDO or TDO, but instead opposes the effects elicited by these enzymes [69]. It could mimic Trp to reverse the inhibition of mTORC1 which is a central regulator for cell growth [70, 71]. There are 17 clinical studies for indoximod registered in *ClinicalTrails* website (https://clinicaltrials.gov) with 12 clinical studies completed, 1 clinical study recruiting, 1 clinical study active, not recruiting, 2 clinical studies terminated (the clinical trial NCT00739609 was lack of enrollment, and the trial NCT03301636 was terminated by the sponsor, not related to efficacy, safety or feasibility), 1 clinical study withdrawn (no results posted). Clinical results indicated that when used as a single agent, indoximod exerted little antitumor efficacy, while combination of indoximod with other therapies including cancer vaccines (Sipuleucel-T/Adenovirus-p53 transduced dendritic cell (DC) Vaccine), checkpoint inhibitors (pembrolizumab/nivolumab/Ipilimumab) and chemotherapy showed markedly enhanced the antitumor efficacy, Indoximod was well-tolerated at doses up to 2000 mg BID [72, 73].

### Epacadostat (INCB024360)

Epacadostat, a Trp competitive inhibitor, has over 1000-fold selectivity to IDO1 over IDO2 or TDO. The half-life of epacadostat is 2.4–3.9 h [74]. In vitro and in vivo (tumor-bearing syngeneic mice) studies showed that epacadostat could reduce tumor growth and promote the proliferation of T cells and NK cells [75]. 55 Clinical studies for epacadostat have been reported, with 7 clinical studies completed, 8 clinical studies recruiting, 18 clinical studies active, not recruiting, 8 clinical studies terminated, 2 clinical studies not yet recruiting, 1 clinical study suspended, 11 clinical studies withdrawn.

Preclinical studies showed that epacadostat and immune checkpoint blockade had a synergy effect, and several clinical trials were initiated to study the combination of epacadostat with either pembrolizumab (NCT03414229, NCT02364076, NCT03291054, etc.), ipilimumab (NCT01604889), nivolumab (NCT02327078, NCT03347123, NCT03348904, etc.). In the trial of NCT03414229, the response rate for melanoma was reported, the objective remission rate (ORR) was 57%, the disease control rate (DCR) was 86%, the ORR and DCR for renal cell carcinoma was 40% and 80%, respectively. In the trial of NCT01604889, the objective response rate was 18%, and stable disease was observed in 26% of the metastatic melanoma patients [76]. In the phase I/II trial of NCT02327078, epacadostat (100 mg BID or 300 mg BID) was combined with nivolumab (240 mg every 2 weeks), the response rate was 62% (6/8 patients receiving 100 mg BID and 25/42 patients receiving 300 mg BID), and toxicities (such as ALT increase, rash and pneumonitis) were observed in 13% of patients receiving epacadostat (100 mg BID) and 48% of patients receiving epacadostat (300 mg BID) [77]. A phase III study (NCT02752074) was designed to investigate the efficacy, safety, and tolerability by combining pembrolizumab with epacadostat or placebo in participants with unresectable or metastatic melanoma, but the results were negative [63]. The disappointing results hampered the development of IDO1 inhibitors, and several phase III trials were terminated and withdrawn. In these terminated and withdrawn clinical trials, 13 studies (NCT03310567, NCT03432676, NCT01685255, NCT03361228, NCT02575807, NCT03463161, NCT03348904, NCT03602586, NCT03238638, NCT03342352, NCT03402880, NCT01604889, and NCT03325465) were due to the reasons including the low enrollment. One study (NCT03832673) was not approved by the Italian Medicines Agency (AIFA). One study (NCT03592407) was due to the safety concerns. One study (NCT03182894) was due to the changes related to the investigational agent.

### BMS-986205

Linrodostat mesylate (BMS-986205), an orally available IDO1 inhibitor, can specifically bind to IDO1, but not IDO2 or TDO. BMS-986205 can reverse the immunosuppression system in cancer patients by inhibiting IDO1 and decreasing the Kyn levels of tumor cells. There are 24 clinical studies for BMS-986205, with 8 clinical studies completed, 11 clinical studies recruiting, 2 clinical studies active, not recruiting, 3 clinical studies withdrawn. The trial (NCT04007588) was withdrawn because of the slow accrual, and another 2 studies (NCT03386838 and NCT03417037) were withdrawn because of the changed business objectives. Several phase I/II studies (NCT03192943, NCT02658890) suggest that combining BMS-986205 with nivolumab is safe and could boost response rates among patients with bladder and cervical cancers (46% and 25%, respectively), and the recommended dose of BMS-986205 was 100 mg for further study [78]. Based on these encouraging results, 13 clinical trials were performed to study the effect of BMS-986205 combined with nivolumab or other drugs such as ipilimumab, relatlimab, chemotherapy, Bacillus Calmette–Guerin (BCG), etc. on non-small cell lung cancer, metastatic or unresectable melanoma, advanced gastric cancer, and other advanced malignant solid tumors [64].

### Navoximod (GDC-0919, NLG-919)

Navoximod is an orally active IDO1 inhibitor. In vitro studies showed that navoximod could restore the T cell function, and in vivo studies suggested that after navoximod treatment, approximately 50% of mice had reduced Kyn levels in plasma. A completed phase I clinical study (NCT02048709) of navoximod monotherapy in recurrent advanced solid tumors showed that 8 (36%) patients and 10 (46%) patients had stable disease and progressive disease, respectively. And clinical adverse events mainly include fatigue (59%), pruritus (41%), nausea (36%), Grade ≥ 3 adverse effects (AEs) occurred in 14/22 patients (64%) [65]. Another completed phase I clinical study (NCT02471846) of navoximod combined with atezolizumab to treat locally advanced or metastatic solid tumors showed that 6 (9%) dose-escalation patients achieved partial response, and 10 (11%) expansion patients achieved partial or complete response. And clinical adverse events mainly include fatigue, rash, and chromaturia. the oral dose of navoximod remains safe and effective up to 800 mg BID [66].

### PF-06840003

PF-0684003, a highly selective and orally bioavailable IDO1 inhibitor, can cross the blood-brain barrier (BBB). It has been reported that PF-0684003 could restore the function of T cells in vitro, and reduce the Kyn levels to enhance the antitumor effect of the checkpoint inhibitors in vivo (syngeneic mouse tumor models) [79]. A phase I study of PF-0684003 (single agent) was performed in patients with recurrent malignant Glioma (NCT02764151) to study the safety, pharmacokinetics/pharmacodynamics, and preliminary efficacy and to determine the maximum tolerated dose (MTD). Patients were divided into four groups: 125 mg once daily (QD, *n* = 2), 250 mg once daily (QD, *n* = 4), 250 mg twice-daily (BID, *n* = 3), 500 mg twice-daily (BID, *n* = 8). Results showed that disease control occurred in eight patients (47%). And the mean duration of stable disease (SD) was 32.1 (12.1–72.3) weeks, four patients experienced serious adverse events (SAEs) [67, 68]. This compound had a prolonged half-life and could cross the blood-brain barrier. But this study was prematurely terminated by the sponsor, and not to pursue marketing approval for the indication of malignant glioma.

### Other inhibitors

There are 2 ongoing phase I clinical trials for KHK2455 (NCT03915405 and NCT02867007). The trial (NCT03915405) is a two-part (dose-escalation, dose-expansion), multicenter, open-label study of KHK2455 in combination with avelumab in adult subjects with urothelial carcinoma. The trial (NCT02867007) is to characterize the safety, tolerability, and determine the maximum tolerated dose (MTD) or the highest protocol-defined dose of KHK2455 administered orally in combination with mogamulizumab (anti-CCR4 antibody) to subjects with locally advanced or metastatic solid tumors [80]. One terminated phase I clinical trial (NCT03343613) for LY3381916 was to evaluate the safety of LY3381916 administered alone or in combination with the anti-PD-L1 antibody LY3300054, and was terminated due to strategic business decision.

### Peptide vaccines for IDO1

Mads Hald Andersen and Inge Marie Svane’s studies have found spontaneous IDO1 specific CD4^+^ and CD8^+^ T cells in tumor infiltrate and peripheral blood of tumor patients, and identified IDO1 specific HLA-A2-restricted epitopes to induce specific CD8^+^ T cells that recognize and kill IDO1-positive cells. Further studies showed that IDO1 specific T cells could also enhance the response of other T cells. A phase I study (NCT01219348) was performed to study the efficiency and safety of the HLA-A2 restricted IDO peptide to treat NSCLC. 15 participants with advanced NSCLC were enrolled. Results showed that the median overall survival (OS) was 25.9 months (> 2 years), progression-free survival (PFS) was 6~7 months, with no grade 3 or 4 toxicities [81, 82]. Based on these promising results, another three clinical trials (NCT01543464, NCT02077114, and NCT03047928) were performed to combine nivolumab or ipilimumab or chemotherapy with IDO peptide to study the synergistic anti-tumor effects [83]. Besides, there are also other peptides that could regulate IDO1 in the study.

## Medicinal chemistry strategies for the development of IDO1 inhibitors in clinical trials

### Epacadostat (INCB024360)

**Fig. 5:**
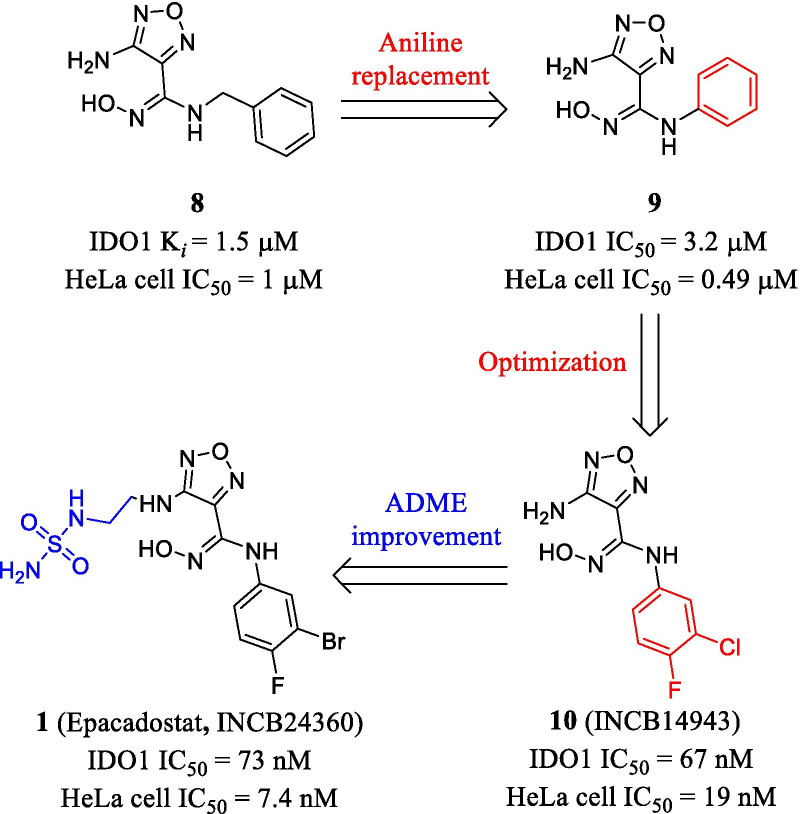
The optimization of epacadostat (INCB24360)

As shown in Fig. 5, the hydroxyamidine chemotype was discovered as a key pharmacophore for IDO1 inhibition. With the assistance of high-throughput screening (HTS), the Incyte Corporation [84] initially identified 4-amino-1,2,5-oxadiazole-3-carboximidamide (compound **8**) as an IDO1 inhibitor (*K*_*i*_ = 1.5 μM), correlative absorption spectroscopy also confirmed direct binding to the heme site. In addition, compound **8** inhibited TDO with an IC_50_ of 10 μM, showing selectivity to IDO1. Subsequent aniline replacement of the benzylamine produced a library of carboximidamides. Intriguingly, compound **9** bearing the phenyl group showed improved cellular potency (HeLa cell IC_50_ = 0.49 μM) than the hit compound **8** (HeLa cell IC_50_ = 1 μM), albeit with slightly decreased enzymatic activity against IDO1 (IC_50_ = 3.2 μM). Among the phenyl substituted derivatives, the *m*-chloro-*p*-fluoro **10** (NCB14943) showed significant improvement in both enzymatic activity and cellular potency (> 100-fold, IDO1 IC_50_ = 67 nM, HeLa IC_50_ = 19 nM) as well as selectivity (IDO2 IC_50_ > 10 μM, TDO IC_50_ > 50 μM). In vivo studies demonstrated that upon subcutaneous administration at 75 mg/kg twice daily, compound **10** caused 50% of tumor growth control (TGC) in naive C57BL/6 mice. Further structural modifications showed that secondary amino substituents at the 3-position of the furazan were preferred over the tertiary amino groups for the activity. It is believed that the secondary amino side chain was exposed to the solvent region of IDO1, thus maintaining preferable biochemical potency. Finally, they obtained the clinical candidate epacadostat (**1**, INCB24360, IDO1 IC_50_ = 73 nM, HeLa IC_50_ = 7.4 nM) with good oral bioavailability across multiple species [85]. As shown in Table 2, epacadostat achieved good oral bioavailability (*F* = 11%, 59%, 33% in rats, dogs, and cynomolgus monkeys, respectively) and a long half-life period (*t*_1/2_ = 2.2 h, 4.9 h, 2.7 h in rats, dogs and cynomolgus monkeys, respectively) without significant loss of the enzyme activity. The PK profiles of **1** in these species demonstrated good exposure (AUC = 1.3 μM·h, 29 μM·h, 9.3 μM·h in rats, dogs, and cynomolgus monkeys, respectively) and moderate permeability (*V*_ss_ = 2.0 L/kg, 0.7 L/kg, 1.8 L/kg in rats, dogs, and cynomolgus monkeys, respectively), which was in good agreement with the vitro clearance data (CL = 1.1 L/h/kg, 0.5 L/h/kg, 0.8 L/h/kg in rats, dogs, and cynomolgus monkeys, respectively). After oral dosing at 30 mg/kg in CT26 model mice, epacadostat suppressed CT26 tumor growth effectively (TGC = 56%). What’s more, epacadostat showed exquisite selectivity over other related dioxygenases (> 1000-fold), and no toxicity in vitro toxicological studies. However, it has been reported that compared with pembrolizumab alone, epacadostat combined with PD-1 monoclonal antibody pembrolizumab did not exhibit significantly improved PFS in the treatment of unresectable or metastatic melanoma [63]. The failure of phase III study of epacadostat posed the need for in-depth insights into the IDO1 pathway in cancers and the rational design of IDO1 inhibitors [86].

**Table 2:** The PK profiles of epacadostat (INCB024360)

Species	CL (L/h/kg)	*V*_ss_ (L/kg)	iv *t*_1/2_ (h)	AUC (μM h)	*F* (%)	*t*_1/2_ (h)
Rat	1.1	2.0	1.4	1.3	11	2.2
Dog	0.5	0.7	3.1	29	59	4.9
Cyno	0.8	1.8	3.3	9.3	33	2.7

CL, plasma clearance; *V*_ss_, volume distribution stead state; *t*_1/2_, half-life; AUC, area under the concentration-time curve; *F*, Oral bioavailability; iv, intravenous injection; Cyno, cynomologous monkeys

**Fig. 6:**
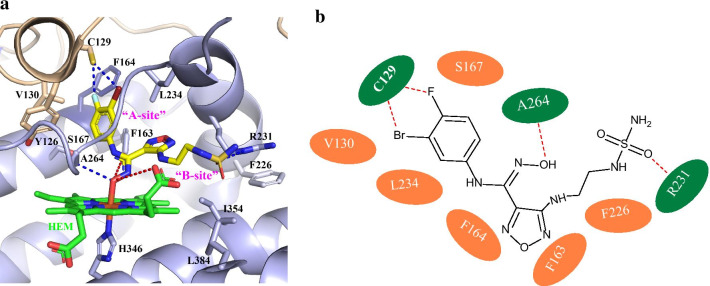
**a** Crystal structure of the IDO1–epacadostat complex (PDB code: 5WN8). The heme site is colored in green. The inhibitor epacadostat is displayed in yellow stick. The H-bonding interactions between epacadostat and the IDO1 protein matrix are indicated by the blue dotted lines, and the intramolecular H-bonds within epacadostat are indicated by the red dotted lines. **b** Schematic illustration of the IDO1–epacadostat interactions.

Syun-Ru Yeh et al. [29] obtained the crystal structure of IDO1 in complex with epacadostat in 2017 (Fig. 6a). The binding model (Fig. 6b) revealed that there was a previously unknown O-based coordination bond between the heme iron and epacadostat, which was simultaneously stabilized by an H-bonding interaction with A264 in the DE-Loop, a fluorine–sulfur contact offered by F and Br atoms with the sulfhydryl of C129 as well as two intramolecular H-bondings of epacadostat. The benzene ring occupied the “A-site” and filled into a hydrophobic pocket formed by a series of residues, including F163, L234, F164, V130, Y126, and S167. On the other hand, the polar side chain of the furazan ring was extended into the “B-site” consisting of F226, R231, I354, L384, and a new H-bond interaction with R231 was observed.

### BMS-986205

BMS-986205 is an inhibitor with a unique dynamic apo-IDO1 binding model [87]. It displayed potent inhibition of kynurenine (kyn) production in HeLa cells (IC_50_ = 1.7 nM) and HEK293 cells (IC_50_ = 1.1 nM) but not TDO (IC_50_ > 20 μM). BMS-986205 showed good oral exposure and efficacy in vivo assays. The pharmacokinetic profiles in rats, dogs, and cynomolgus monkeys were summarized in Table 3. After an intravenous administration of 0.5 mg/kg, the corresponding clearance, *V*_ss_, half-life and bioavailability in rats were 27 mL/min/kg, 3.8 L/kg, 3.9 h and 4%, respectively. The results in dogs at 0.5 mg/kg were 25 mL/min/kg, 5.7 L/kg, 4.7 h and 39%, respectively. In cynomolgus monkeys with the same doses as rats, the results were 19 mL/min/kg, 4.1 L/kg, 6.6 h and 10%, respectively.

**Table 3:** The PK profiles of BMS-986205

Species	CL (mL/min/kg)	*V*_ss_ (L/kg)	*t*_1/2_ (h)	*F* (%)
Rat	27	3.8	3.9	4
Dog	25	5.7	4.7	39
Cyno	19	4.1	6.6	10

CL, plasma clearance; *V*_ss_, volume distribution stead state; *t*_1/2_, half-life; *F*, oral bioavailability; Cyno, cynomologous monkeys

**Fig. 7:**
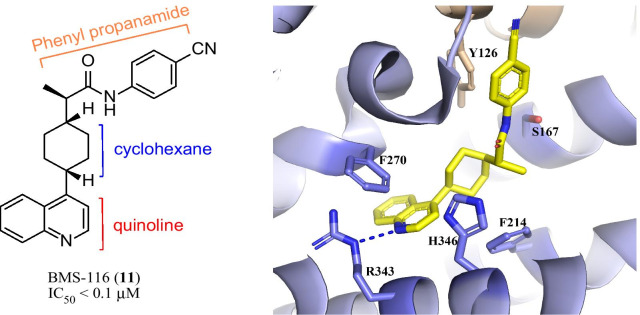
The binding mode of BMS-116 with IDO1 (PDB code: 6AZW). The inhibitor BMS-116 is displayed in yellow stick. The H-bonding interaction is indicated by the blue dotted lines

The X-ray crystallographic structure of BMS-116 (**11**, IC_50_ < 0.1 μM, Fig. 7, PDB: 6AZW) [88], an analog of BMS-986205, revealed a putative substrate-binding site, where the cyanophenyl group in BMS-116 bond with Y126 via π–π interactions and had hydrogen-bond interaction with S167. The quinoline ring occupied an additional hydrophobic pocket formed by F270, F214, H346 and R343, and formed “edge-to-face” interaction with F270 and H-bonding with R343. In addition, the cyclohexane group also played an auxiliary role in guiding the quinoline ring to the new binding pocket. The unique binding mode of BMS-116 may provide a structural basis for the design of new IDO1 inhibitors.

### PF-06840003

**Fig. 8:**
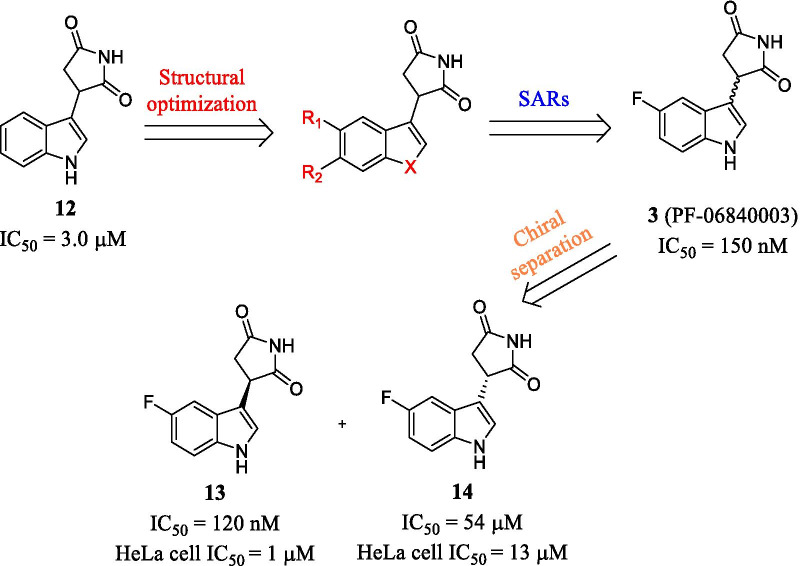
The discovery of PF-06840003

Among indoles series, PF-06840003 (EOS200271, **3**), developed by iTeos and Pfizer, is a representative non-heme-binding IDO1 inhibitor with high selectivity and excellent pharmacokinetics [59]. As shown in Fig. 8, compound **12** showed moderate potency against IDO1 (IC_50_ = 3.0 μM), while showing no activity against IDO2 or TDO (IC_50_ > 200 μM). Introducing halogens to C-5 resulted in a significant increase in activity (**3**, IC_50_ = 150 nM), whereas other substitutions led to decreased potency and increased tolerance. Among two enantiomers of **3**, only compound **13** inhibited IDO1 potently (IC_50_ = 120 nM) and exhibited good antiproliferative activity against HeLa cells (IC_50_ = 1.0 μM). As shown in Table 4, compound **3** exhibited a low plasma clearance in rat (3.7 mL/min/kg) and human (0.64 mL/min/kg), and moderate plasma clearance in the dog (24.3 mL/min/kg) with a low volume of distribution in these species after intravenous dose of 1 mg/kg (0.639 L/kg, 0.957 L/kg, 1.1 L/kg, respectively), and the t_1/2_ of **3** in rats, dogs as well as human was 2.42 h, 0.84 h, 19 h, respectively. Oral bioavailability was 94% in rats, 64% in human but only 18.6% in dogs, the high area under the concentration-time curve (AUC) in rats and low AUC in dogs were also consistent with measured bioavailability. Therefore, based on the excellent PK profiles, the racemate **3** was selected for pre-clinical and clinical studies.

**Table 4:** The PK profiles of PF-06840003

Species	CL (mL/min/kg)	*V*_ss_ (L/kg)	*t*_1/2_ (h)	*F* (%)	AUC (ng h/mL)
Rat	3.7	0.639	2.42	94.0	6940
Dog	24.3	0.957	0.840	18.6	237
Human	0.64	1.1	19	64	ND

CL, plasma clearance; *V*_ss_, volume distribution stead state; *t*_1/2_, half-life; *F*, Oral bioavailability; AUC, area under the concentration-time curve

**Fig. 9:**
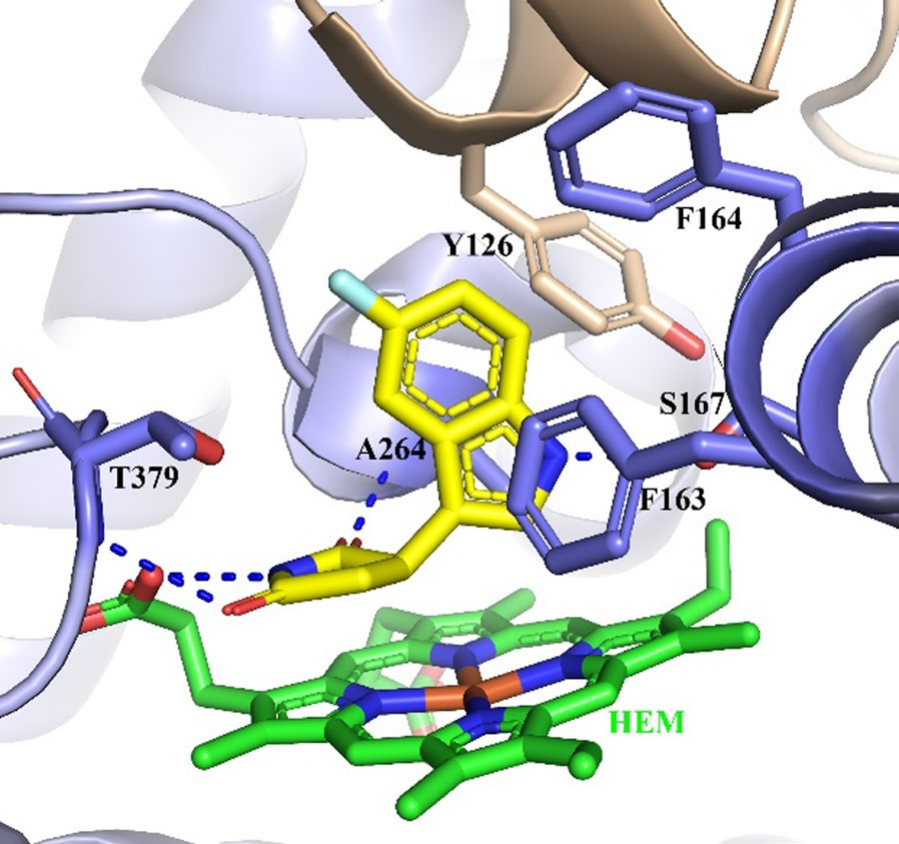
The co-crystal structure of the complex IDO1-PF06840003 (PDB code: 5WHR). The heme site is colored in green. The inhibitor PF06840003 is displayed in yellow stick. The H-bonding interactions between PF06840003 and the IDO1 protein matrix are indicated by the blue dotted lines

The binding mode of the **3**-IDO1 complex (PDB code: 5WHR) is illustrated in Fig. 9 [59]. There was no direct interaction between **3** and the heme, despite the indole ring occupied the lipophilic pocket. The heteroatoms formed H-bonding interactions. The indole NH hydrogen bond to the side chain of S167, the succinimide NH interacted with the heme carboxylic acid, additional two H-bond interactions were observed between two carbonyls and the main chain of A264 and T379. Moreover, the indole aromatic core also formed hydrophobic contacts with several aromatic residues (Y126, F163, F164). The formation of these hydrogen-bond networks and the hydrophobic interactions between the indole ring and the binding pocket significantly contribute to the activity of PF06840003.

### Navoximod (GDC-0919, NLG-919)

**Fig. 10:**
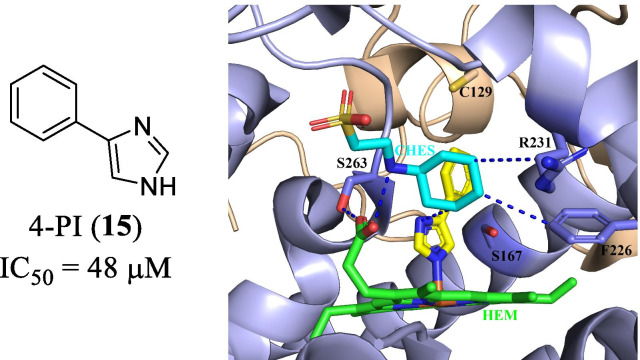
The active site of the IDO1/4-PI complex (PDB code: 2D0T). The heme site is colored in green. The 4-PI is displayed in yellow stick, and the CHES is highlighted in cyan. The H-bonding interactions are indicated by the blue dotted lines

In 1989, Sono M et al. [30] reported the 4-phenyl-imidazole (4-PI, **15**, IC_50_ = 48 μM, Fig. 10) as a weak noncompetitive inhibitor. The binding to the heme iron of IDO1 was subsequently confirmed by the co-crystal structure of 4-PI/IDO1 (PDB code: 2D0T). The co-crystal structure revealed some key binding modes: (1) the *N*-cyclohexyl-2-aminoethanesulfonic acid (CHES, highlighted in cyan) occupied the active site entrance region and its amino group and the side chain of S263 formed hydrogen-bond interaction with heme 7-propionic acid group, respectively; (2) the H-bonding interactions were observed between CHES molecule and F226, R231 as well as the N-atom of the imidazole ring of 4-PI; (3) the distal heme pocket was a coordination site for the sixth external ligand (the imidazole ring).

**Fig. 11:**
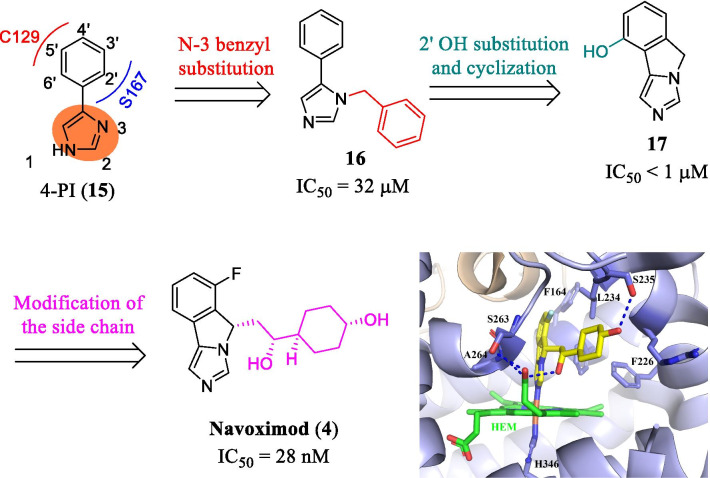
The development of navoximod (PDB code: 6O3I). The heme site is colored in green. The inhibitor navoximod is displayed in yellow stick. The H-bonding interactions between Navoximod and the IDO1 protein matrix are indicated by the blue dotted lines

Based on the binding interactions with IDO1, further optimization led to the discovery of a potent, orally bioavailable, and efficacious clinical candidate navoximod (NLG919, **4**, Fig. 11) [89, 90]. The docking revealed that substitution on the imidazole ring of 4-PI was tolerable, and substitutions on the N-3 position of the imidazole ring would occupy the active site entrance containing a CHES buffer molecule. Compared to **14**, the *N*-benzyl substituted derivative **15** showed slightly improved activity (IC_50_ = 32 μM). The crystal structure of 4-PI bound to IDO1 (Fig. 10) revealed that the phenyl ring was in close proximity to S167 and C129. Thus, modifications on the phenyl ring may enhance the H-bonding interactions with S167. Researchers from the Newlink Company designed compound **17** (IC_50_ < 1 μM) through connecting N-3 position and 2′ position, which occupied the active site and formed favorable protein–ligand interactions. Following the tricyclic scaffold, they finally obtained another imidazoisoindole-based potent and orally bioavailable clinical candidate **4** (IDO1 IC_50_ = 28 nM). Compound **4** eliminated IDO1-induced T cell suppression and restored T cell responses with an EC_50_ value of 80 nM. Regrettably, the overall response rate of navoximod combined with atezolizumab in all types of tumors was only 10%. To corroborate the binding mode of navoximod, Kumar et al. [90] determined the structure of the IDO1/navoximod complex (PDB code: 6O3I, Fig. 11). The complex demonstrated that the imidazoisoindole ring of **4** coordinated with heme iron, which significantly contributed to the improved potency. And the extensive hydrophobic interactions were observed between the cyclohexane group with surrounding residues F163, F164, L234, etc. Notably, the middle hydroxy group served as an H-bonding donor to form an intramolecular hydrogen bond with the nitrogen atom of the isoindole core, which played an important role in stabilizing the conformation, enhancing the activity and improving the physicochemical properties of the compound. In addition, the terminal hydroxyl formed an H-bonding interaction with S235, and another H-bonding interaction was observed between S263 and the heme molecule. Overall, these observed interactions are responsible for the excellent inhibitory activity and selectivity of **4**.

The pharmacokinetics of **4** were also studied in rats, mice and dogs following 10 mg/kg administration (Table 5). Compound **4** exhibited an t_1/2_ of 1.44 h, 3.9 h, 4.8 h in rats, mice and dogs, respectively, the relevant plasma clearance was 44.6 mL/min/kg, 27.5 mL/min/kg, 53.7 mL/min/kg, respectively. After oral administration of 50 mg/kg for 1 h, the concentration of compound **4** in plasma (1560 ng/mL in rats, 2257 ng/mL in mice, 1400 ng/mL in dogs) was higher than that in other organs. In mice, compound **4** showed a higher bioavailability of 69% than that in rats (41.5%) and dogs (22.3%), the results were also consistent with measured AUC value of 6132 ng h/mL (only 3930 ng h/mL in rats and 3127 ng h/mL in dogs). In short, compound **4** had a favorable drug-like profile as a clinical candidate.

**Table 5:** The PK profiles of navoximod

Species	CL (mL/min/kg)	*C*_max_ (ng/mL)	*t*_1/2_ (h)	*F* (%)	AUC (ng h/mL)
Rat	44.6	1560	1.44	41.5	3930
Mice	27.5	2257	3.9	69.0	6132
Dog	53.7	1400	4.8	22.3	3127

CL, Plasma clearance; *C*_max_, peak concentration; *t*_1/2_, half-life; *F*, Oral bioavailability; AUC, area under the concentration-time curve

### The effector inhibitor indoximod (1-methyl-*D*-tryptophan, 1MT, NLG-8189) and its prodrug NLG802

**Fig. 12:**
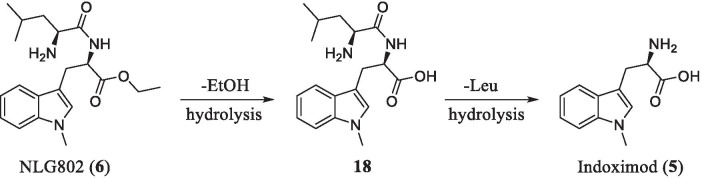
Metabolic transformations of NLG802 in human hepatocytes

Trp, the endogenous ligand, had a weak inhibitory activity against IDO1 [91]. *DL*-1MT is the first reported IDO1 inhibitor (*K*_*i*_ = 34 μM) as the Trp mimetic. After chiral resolution, *L*-1MT showed a weak inhibitory activity (*K*_*i*_ = 19 μM), but *D*-1MT (indoximod, **5**) did not (Fig. 12). Intriguingly, compound **5** had better antitumor activity in combination with chemotherapy drugs [92]. It is generally believed that **5** can regulate the transmembrane transport of Trp and modulate the downstream signaling pathway of IDO1 as an IDO1 bypass inhibitor. In addition, **5** could also up-regulate the co-regulatory receptor inducible co-stimulator (ICOS) of effector T cells as the Trp mimetic and exert an additional immunomodulatory effect.

Additionally, indoximod had a linear PK profile below 1200 mg/dose while increasing doses above 1200 mg/dose would cause a non-linear increase in exposure, which meant that higher concentrations of indoximod would be required to expand the pharmacodynamic effects due to the limited bioavailability. Therefore, Mautino et al. [72] synthesized a series of indoximod prodrugs including NLG802 (**6**) and assessed their pharmacokinetics in rats as well as in cynomolgus monkeys. As shown in Fig. 12, NLG802 was preliminarily metabolized to the intermediate **18** via hydrolysis, which was then converted to Leu and indoximod. In mice models, compared with the equivalent oral dose of **5**, the maximum concentration (*C*_max_) of **6** increased by a factor of 2–4 in the range of 143–1147 μM/kg (Table 6), and observed daily exposure (AUC_0–24_) of **6** also increased 2 and threefold at 287 and 143 μM/kg, respectively, but the values became comparable above 573 μM/kg, which suggested that there was a certain saturation in PK at high dose level. In general, **6** was rapidly absorbed and metabolized into indoximod in vivo (*t*_1/2_ = 2.2–3.2 h). Furthermore, the toxicity studies revealed no adverse effect level (NOAEL) with 120 mg/kg/dose bid, which was equivalent to 3200 mg/kg/dose bid in human. In conclusion, the prodrug **6** increased drug exposure and could bring additional therapeutic benefits to patients.

**Table 6:** Comparison of pharmacokinetics after a single dose of 5 or 6 in mice

Compound	Dose (μM/kg)	*C*_max_ (μM)	AUC_0–24_ (μM h)	*t*_1/2_ (h)
Indoximod (**5**)	143	9.3 ± 2.2	36.4 ± 9	3
	287	17.2 ± 3.7	77.7 ± 21.6	2.8
	573	26 ± 11.1	153.6 ± 79.2	3.1
	1147	51.7 ± 25.4	348.2 ± 115.2	3.4
NLG802 (**6**)	143	35 ± 25.3	105.4 ± 68.5	2.9
	287	68.9 ± 3.9	142.3 ± 64.6	2.2
	573	65.7 ± 34.1	150.8 ± 76.1	3.1
	1147	101.4 ± 38.9	374.3 ± 180.7	3.2

*C*_max_, maximum concentration observed in plasma; AUC_0–24_, area under the plasma concentration-time curve from 0 to 24 h of quantifiable concentration; *t*_1/2_, half-life

### Other clinical inhibitors

In 2018, Dorsey et al. [93] discovered the indoline derivative LY3381916 (**7**) as a selective and potent inhibitor of IDO1 (IDO1 IC_50_ = 7 nM, IDO2 IC_50_ > 20 μM). They identified that **7** bonds to synthesized apo-IDO1 lacking heme, whereas **7** did not inhibit mature heme-bound IDO1, the novel mechanism suggests that the inhibition effect of IDO1 in tumors required the turn-over of mature heme-bound IDO1. LY3381916 has been studied in phase I clinical trial since 2017, and its therapeutic effect could be strengthened when combined with PD-L1 inhibitors, but this trial (NCT03343613) was terminated in 2020. Additionally, LY3381916 is safely administered as monotherapy, the recommended dose for combination with PD-L1 in expansion cohorts is 240 mg (QD). In addition to the aforementioned inhibitors, other small molecules IDO1 inhibitors, including SHR9146 (the dual IDO/TDO inhibitor, NCT03208959), KHK2455 (NCT03364049) and IO102 (NCT04445064), are also at different stages of clinical trials. The chemical structures of these inhibitors and related data have been undisclosed.

## Conclusions and perspectives

Cancer immunotherapy has been recognized as an effective strategy to eliminate tumor cells. IDO1 plays a pivotal role in catalyzing the Trp–Kyn pathway, and its overexpression in multiple tumor types is believed to result in either complete or local suppression of the immune system via depleting local Trp storage and accumulating downstream metabolites. Thus, IDO1 has been recognized as an attractive immunomodulatory target. Consequently, multiple IDO1-targeting therapeutic options (e.g., inhibitors, peptide vaccines, combination with anti-PD1 antibody, PROTACs) for various cancers are currently under development.

On the basis of structural features of IDO1, many small-molecule inhibitors that could coordinate with the heme iron and occupy both the hydrophobic “Pocket A” and the extended conserved “Pocket B” have been developed, these structural features may provide basis for further structure-based design of IDO1 inhibitors. To date, some IDO1 inhibitors have entered clinical trials for treating various solid tumors. IDO1 inhibitors in combination with the anti-PD1 antibody have also displayed better cooperativity, which could overcome drug resistance and maximize survival benefits of patients. However, the failure of phase III study of epacadostat is the key turning point in the development of IDO1 inhibitors, it not only dismayed the research community but prompted several pharmaceutical companies to suspend or terminate relevant ongoing trials. Therefore, insights into the IDO1 inhibition mechanism and rational trial design may be a priority in IDO1-targeting small-molecule drug discovery.

The proteolysis-targeting chimeras (PROTACs) is an emerging strategy to degrade oncogenic proteins via the ubiquitin-proteasome pathway, a few of PROTACs molecules have been reported, and some of them have advanced into clinical trials, showing promise for cancer therapy [94–97]. To date, the first PROTAC-based IDO1 degrader has been reported [27], which conjugates the IDO1 inhibitor epacadostat to the CRBN ligand lenalidomide through a hydrophilic linker. The degrader showed effective degradation of IDO1 (*D*_max_ = 93%, DC_50_ = 2.84 μM) in HeLa cells and improved inhibitory activity against HER2 CAR-T cells. The discovery of this IDO1 degrader shows a novel therapeutic option, albeit with moderate degradation potency, more potent IDO1 degraders will be developed in the near future and may find clinical applications in the clinic.

Although significant progress has been made so far, some issues still exist, including: (1) the activation of AhR by IDO1 inhibitors could induce pro-carcinogenic effects in several human cancers and may be associated with poor prognosis, but it remains unknown whether the prolonged AhR activation affects cancer progression; (2) IDO1 inhibitors could mimic Trp as fake nutritional signals and reactivate the mTOR activity inhibited by Trp depletion, which may cause artificial antitumor efficacy of these inhibitors. Notably, the activation of mTOR is also capable of reactivating the T-cell function and may contribute to overcome the tumor immune escape; (3) It is worth considering whether patient populations in trials need to be stratified according to the expression level of IDO1; and (4) Due to the potential compensatory mechanism of Trp involving IDO2 or TDO, the IDO/TDO dual inhibition may be regarded as an improved approach for inhibiting the Trp–Kyn pathway. Hence, an in-depth understanding of the roles of IDO1 in cancer immunology and the search for novel IDO1 inhibitors as well as alternative targeted IDO1 therapies are still needed.

## Data Availability

Not applicable.

## Abbreviations

AhR: Aryl hydrocarbon receptor
APCs: Antigen-presenting cells
Bin1: Bridging integrator 1
COX-2: Cyclooxygenase
CHES: The *N*-cyclohexyl-2-aminoethanesulfonic acid
CTD: C-terminal domain
DAMPs: Damage-associated molecular patterns
DCs: Dendritic cells
DEL: DNA-encoded library
ECs: Endothelial cells
GCN2: General control over nonderepressible 2
GITRL: Glucocorticoid-induced TNF receptor-ralated protein
hIDO1: Human IDO1
HEM: The heme site
HTS: High throughput screening
ICOS: Inducible co-stimulator
IDO1: Indoleamine-2,3-dioxygenase-1
IL: Interleukin
IFN: Interferon
IFNR: IFN receptor
IRF1: IFN regulatory factor 1
ISRES: IFN-γ stimulated response elements
ISX: Intestine-specfic homeobox
JAK: Janus kinase
KP: Kynurenine pathway
Kyn: Kynurenine
mTOR: The mammalian target of rapamycin
NAD^+^: Nicotinamide adenine dinucleotide
NKs: Natural killer cells
NPs: Natural products
NTD: N-terminal domain
PAMPs: Pathogen-associated molecular patterns
PEG: Polyethylene glycol
PGE2: Prostaglandin E2
PGER: PGE2 receptor
PI3K: Phosphoinositide 3-Kinase
PKCδ: Protein kinase C δ
PROTAC: Proteolysis targeting chimera
SAR: Structure–activity relationship
SBDD: Structure-based drug design
STAT: Transducer and activator of transcription
TAAs: Tumor-associated antigens
TDO: Tryptophan 2,3-dioxygenase
T_eff_: Effector T cells
TGC: Tumor growth control
TGF: Transforming growth factor
TGFR: TGF receptor
TLR: Toll-like receptor
TMB: Tumor mutation burden
TNF-α: Tumor necrosis factor α
Tregs: Regulatory T cells
Trp: Tryptophan
UPS: Ubiquitin/proteasome system
4-PI: 4-Phenylimidazole
